# Consumer knowledge of and engagement with traditional takeaway and dark kitchen food outlets

**DOI:** 10.3310/nihropenres.13735.2

**Published:** 2025-03-10

**Authors:** Lucie Nield, Helen Martin, Claire Wall, Jo Pearce, Rachel Rundle, Simon Bowles, David Harness, Jordan D. Beaumont

**Affiliations:** 1 Sheffield Business School, Sheffield Hallam University, Sheffield, England, S1 1WB, UK

**Keywords:** Consumer behaviour, delivery-only kitchen, ghost kitchen, virtual kitchen

## Abstract

**Background:**

Dark kitchens – delivery-only food outlets operating through digital technology platforms – are a contemporary addition to the food environment. Some concerns have been raised around the ability for local authorities to identify and regulate these businesses, with growing concern around the nutritional quality of foods, food safety practices and impact on the local food environment. The present work explores consumer understanding of and engagement with dark kitchen and traditional takeaway establishments.

**Methods:**

Healthy adults living in the United Kingdom completed an online survey comprising of questions measuring demographics, engagement with takeaways and dark kitchens, purchasing behaviours and decision making, and knowledge and understanding around dark kitchens. Data were analysed using descriptive statistics.

**Results:**

In total, 2,023 participants (46.3 ± 16.7 years) completed the survey. Forty percent purchased a takeaway at least weekly, often through aggregator applications (e.g., Just Eat, Deliveroo). Food was mainly purchased as a treat (79.3%), for enjoyment of the food or taste (60.8%) and for convenience (58.2%). When ordering, consumers considered the taste (88.1%), quality (83.5%), value for money (77.8%), and familiarity with (68.1%) and reputation of the business (60.0%). Only 24.7% of participants had heard of dark kitchens and 9.1% had knowingly purchased from one. After reading a working definition, 54.9% said they would purchase from a dark kitchen, but most would want to know explicitly that they were ordering from these businesses. A major concern when purchasing food from a dark kitchen or takeaway outlet was trust in the food safety and hygiene standards.

**Conclusion:**

Consumers are unfamiliar with dark kitchens and are not aware of or confident in identifying these businesses. This confusion and concerns around food safety mean dark kitchens are often viewed negatively. Consumers would prefer more transparency in where their foods are being prepared to allow for more informed decision-making.

## Plain Language Summary

This study used an online survey to ask people living in the UK about their use of takeaways and what information and understanding they had about a type of delivery-only takeaway called ‘dark kitchens’. This work is important because dark kitchens are very common in the UK and are a new type of food outlet which are less well-researched. This is the first study to ask people living in the UK what they know and think about dark kitchens. The study found that only one-quarter of people had heard of the term dark kitchens and that most people (91%) had never knowingly used one to purchase food. People were worried about the quality and safety of the food and whether the business was legal. People would like to know whether the food they buy comes from a dark kitchen or not so that they can use this information when deciding where to buy their food from.

## Introduction

Dark kitchens, also called ghost or cloud kitchens, are delivery-only ‘virtual’ commercial food spaces that do not have a customer-facing storefront and operate largely through third-party aggregator platforms (e.g., Just Eat, Uber Eats, Deliveroo). They are a contemporary addition to the food environment in the United Kingdom (UK), likely finding popularity during the COVID-19 pandemic ( Keeble *et al.*, 2023). Operating at relatively low-cost within urban environments with easy access to consumers and potential for large geographical reach ( Rinaldi *et al.*, 2022), dark kitchens offer a lucrative business model ( de Souza *et al.*, 2022; Food Standards Agency, 2022) within a rapidly growing market ( Statista, 2022). Indeed, the worldwide market size is predicted to increase to $177.85 billion by 2032, an increase of more than $122 billion since 2022 ( Statista, 2024).

The growth of dark kitchens presents additional complexity when evaluating the place-based food environment and potential impact on health ( Keeble *et al.*, 2020; Rinaldi *et al.*, 2022), compounded by lack of clarity of how many exist and how to define them. There is a need to understand the complexity of the changing foodscape from both a professional and consumer awareness perspective. However, dark kitchens are particularly difficult to monitor and regulate ( Food Standards Agency, 2022) as the number, type and impact is largely unknown. The lack of clarity in nutritional quality of foods offered by many of these businesses ( Fernandez & Raine, 2021; Keeble *et al.*, 2020), and potential for poor food safety practices ( Crawford & Benjamin, 2019) are cause for concern, and best practice is yet to be established.

Increasingly, consumers are keen to understand where their food is coming from, with recent purchasing trends related to ingredient provenance and transparency of the food supply chain leading to improved consumer trust and brand loyalty which drives intention to purchase ( Cai *et al.*, 2022; Moralez, 2019). While there is some evidence of consumer attitudes towards dark kitchens (e.g., Pookulangara *et al*., 2023), there is limited understanding of consumer awareness of dark kitchens and how the dark kitchen sector influences consumer decision making and purchase behaviours. However, the online third-party aggregator platforms do provide a level of data transparency for the consumer by providing food hygiene ratings and other purchaser reviews on their platforms which are easy to access, to allow the consumers to make more informed food choices.

### Aim and objectives

This study aimed to explore consumers current awareness of and engagement with traditional and dark kitchen takeaway outlets, and specifically their knowledge of dark kitchens. The study objectives were:

- Establish consumer usage of traditional and dark kitchens takeaways
- Understand how consumers currently perceive risks and benefits of dark kitchens, and how this changes their engagement.

## Methods

### Patient and Public Involvement (PPI)

During project development, the research team consulted with individuals from local authorities across Yorkshire and the Humber and from the Public Involvement in Research Group (PIRG) – a PPI network at Sheffield Hallam University. Two PIRG members were included in the project steering committee and had oversight of the project. These individuals contributed to project development.

### Ethics

All procedures underwent independent review by the Sheffield Hallam University research ethics committee and the application was approved on 06 February 2024 (ethics approval number: ER61546845). All participants provided written, informed consent. Procedures adhered to the Declaration of Helsinki.

### Participants

Participants were healthy adults (18 years of age or older) living in the UK. A representative sample of the national population was recruited through the Prolific platform; sample size was stratified based on sex, age and ethnicity in line with census data ( Office for National Statistics, 2024). The study aimed to recruit a minimum of 2,000 participants.

### Study design, materials and analysis

The study involved a brief online cross-sectional survey through Qualtrics, lasting approximately 15 minutes. The questionnaire included a series of standardised demographic questions aligned with census question format, which included age, gender, ethnicity, household composition, and total pre-tax household income. Participants’ engagement with takeaways and dark kitchens (e.g., typical spend per week, frequency of purchase), purchasing behaviours and decision making (e.g., reason for purchase, influential factors when considering purchasing a takeaway, importance of food safety and hygiene measures), and knowledge and understanding around dark kitchens (e.g., prior knowledge of dark kitchens, awareness of dark kitchens within local area) were measured primarily through quantitative, closed-ended questions with further open-ended questions requesting additional information. Participants responded to up to 33 questions in total. The survey was designed with input from Patient and Public Involvement (PPI) and stakeholder groups, and was refined and piloted prior to the study to ensure data collected answered the research aims. A copy of the survey is available via the Open Science Framework ( https://doi.org/10.17605/OSF.IO/6SWBK). Descriptive statistics were used to analyse data and where available summaries of qualitative data are provided to support descriptive statistics. The study procedures were pre-registered on Research Registry (unique identifying number: researchregistry10007). The study is reported using STROBE guidelines.

## Results

In total, 2,023 participants responded to the online questionnaire between April and June 2024. Mean age of the participants was 46.3 ± 16.7 years (range: 18 to 91 years). Further demographic characteristics are displayed in Table 1. A representative sample of the UK population were recruited, with demographic profiles (for age, sex and ethnicity) aligning with the most recent census data ( Office for National Statistics, 2024). Most participants (n = 1,990, 98.5%) were above the income tax threshold at the time of completion of £12,571 ( Table 2). Prior to being involved in the study, only 24.7% of respondents had heard of dark kitchens, mainly through their interactions on social media and online platforms, and only 9.1% had knowingly purchased from a dark kitchen restaurant. However, based on reading a working definition of dark kitchens, 54.9% of consumers said that they would purchase from a dark kitchen. The remaining participants would not purchase from a dark kitchen as they would prefer to see the establishment, know that they could see where their food was being prepared, or were dubious about hygiene standards and legality of such establishments. The majority (65.9%) of respondents would want to know explicitly whether they were ordering food from a dark kitchen.

**Table 1.** Participant demographic characteristics (n = 2,023).

*Gender*	*n*	*%*
Female	1,017	50.2
Male	985	48.7
Genderqueer / Gender non-conforming	9	0.4
Non-binary	2	0.1
Transgender man	1	0.1
Transgender woman	1	0.1
Declined to state	8	0.4
*Ethnicity*	*n*	*%*
English, Welsh, Scottish, Northern Irish or British	1,604	79.3
Any other White background	95	4.7
Indian	50	2.5
African	42	2.1
Any other Asian background	34	1.7
Chinese	33	1.6
Pakistani	32	1.6
Irish	30	1.5
White and Black Caribbean	24	1.2
Caribbean	17	0.8
Bangladeshi	12	0.6
Any other Mixed or multiple ethnic background	12	0.6
Not listed or other	10	0.5
Arab	8	0.4
White and Asian	7	0.4
Any other Black, Black British, or Caribbean background	7	0.4
White and Black African	6	0.3
Gypsy or Irish Traveller	0	0.0
Roma	0	0.0
*Employment status*	*n*	*%*
In full-time employment	771	38.1
Not in paid employment (e.g., homemaker, retired, disabled)	386	19.1
In part-time work	279	13.8
Unemployed / Job seeking	84	4.2
Other	59	2.9
Declined to state	444	21.9
*Student status*	*n*	*%*
No	1,537	80.0
Yes	162	8.0
Declined to state	324	16.0
*Index of Multiple Deprivation (total n = 1,866)*	*n*	*%*
Quintile 1	288	15.4
Quintile 2	387	20.7
Quintile 3	418	22.4
Quintile 4	379	20.3
Quintile 5	394	21.1

**Table 2.** Annual pre-tax household income (n = 2,021).

*GBP (£)*	*n*	*%*
£4,999 or less	38	1.9
£5,000 to £12,570	94	4.7
£12,571 to £20,999	201	9.9
£21,000 to £30,999	372	18.4
£31,000 to £40,999	314	15.5
£41,000 to £50,999	282	14.0
£51,000 or more	720	35.6

Many participants purchase a takeaway at least once a week (n = 748, 31.7%) ( Table 3), with a typical spend of up to £20 per purchase ( Table 4). Purchases were often made using aggregator/third party mobile applications such as Just Eat, Deliveroo and Uber Eats (51.3%) or brand-specific mobile application (e.g., through the McDonalds app) (28.6%). Most of these takeaway purchases (n = 1,911, 67.9%) were for adults within the household, whilst 16.7% (n = 471) of purchases were reported to be for children and young people in the household. A further 15.3% (n = 429) of purchases were for people outside of the household such as family, visitors and friends.

**Table 3.** Frequency of takeaway purchase (n = 2,023).

	*n*	*%*
Every day	1	0.1
5 to 6 times per week	6	0.3
3 to 4 times per week	48	2.4
Once or twice a week	693	34.3
Once a fortnight	503	24.9
Once a month	392	19.4
Less than once a month	326	16.1
Never	52	2.6
Declined to state	2	0.1

**Table 4.** Total household spend (GBP, £) on food per week (n = 2,023).

Spend on food shopping *	*n*	*%*
£0.01 to £19.99	20	1.0
£20.00 to £39.99	196	9.7
£40.00 to £59.99	354	17.5
£60.00 to £79.99	435	21.5
£80.00 to £99.99	422	20.9
£100.00 to £149.99	447	22.1
£150.00 to £199.99	108	5.3
£200.00 or more	37	1.8
Declined to state	4	0.2
*Spend on eating out ^†^*	*n*	*%*
£0.00	297	14.7
£0.01 to £19.99	787	38.9
£20.00 to £39.99	510	25.2
£40.00 to £59.99	249	12.3
£60.00 to £79.99	94	4.6
£80.00 to £99.99	47	2.3
£100.00 to £149.99	26	1.3
£150.00 to £199.99	5	0.3
£200.00 or more	5	0.3
Declined to state	3	0.1
*Spend on takeaways/dark* *kitchens*	*n*	*%*
£0.00	326	16.1
£0.01 to £19.99	868	42.9
£20.00 to £39.99	590	29.2
£40.00 to £59.99	164	8.1
£60.00 to £79.99	39	1.9
£80.00 to £99.99	17	0.8
£100.00 to £149.99	12	0.6
£150.00 to £199.99	3	0.1
£200.00 or more	2	0.1
Declined to state	2	0.1

* This includes foods purchased at shops, supermarkets, markets, etc., but does not include food eaten out of the home (e.g., takeaways, eating at a restaurant). † This includes eating out at restaurants, cafes and canteens, but does not include takeaway establishments.

The most common reasons that people purchased from a takeaway were for a treat (79.3%), for enjoyment of the food or taste (60.8%) and for convenience (58.2%) ( Table 5). Other reasons for purchasing a takeaway include being too tired or ill to cook, it was too late to start cooking, or to purchase foods that they could not prepare in the home (e.g., do not have the equipment or ingredients available). When ordering a takeaway, the most common considerations were the taste of the food (88.1%), quality of the food (83.5%), value for money (77.8%), familiarity with the business (68.1%) and reputation of the business (60.0%) ( Table 6). Participants were less concerned with ethical standards of the food or business (9.1%), sustainability of the food or business (4.7%), provenance of ingredients (4.6%) and allergen control (4.2%). Other factors considered included availability of vegan, halal and gluten-free options.

**Table 5.** Reasons for ordering food from a takeaway or dark kitchen (n = 1,966).

	*n*	*%*
It is a treat	1,560	79.3
I enjoy the food/I enjoy the taste	1,196	60.8
It is convenient	1,144	58.2
For a special occasion (e.g., birthday)	727	37.0
There is a large variety of foods I wouldn't cook at home	388	19.7
I do not have enough time to cook	379	19.3
It is part of my/our routine (e.g., get a takeaway every Friday)	298	15.2
I want to try something new	264	13.4
It is often an impulsive purchase (e.g., I've seen an advert)	252	12.8
Other	93	4.7
I can't cook/I am not a confident cook	56	2.8
It is cheaper to purchase takeaway food than cook food in the home	32	1.6
I have limited access to other sources of food	26	1.3

**Table 6.** Factors considered when purchasing from a takeaway or dark kitchen (n = 1,972).

	*n*	*%*
Taste of the food	1,737	88.1
Quality of the food	1,646	83.5
Cost or value for money	1,535	77.8
Familiarity with the takeaway/restaurant	1,342	68.1
Reputation of the takeaway/restaurant	1,183	60.0
Speed/convenience of ordering	1,079	54.7
Location or proximity of the business	1,039	52.7
Trustworthiness of the takeaway/restaurant	995	50.5
Food hygiene rating of the business	932	47.3
General cleanliness standards of the business	863	43.8
Timing of delivery	858	43.5
Healthiness or nutritional quality	260	13.2
Ethical standards of the food or business (e.g., animal welfare)	180	9.1
Sustainability of the food or business	93	4.7
Where ingredients are sourced (i.e., provenance)	90	4.6
Allergen control	83	4.2
Other	32	1.6

The food hygiene rating of a business was considered by 47.3% of participants ( Table 6); in line with this, 56.0% (n = 1,105) of participants reported they checked food hygiene ratings before they order from a takeaway. Of these, 93.7% (n = 1,033) stated the food hygiene rating would affect their willingness to purchase from the business, with many participants stating they would only order if the business received a rating of at least 4 out of 5. Low ratings were of concern due to perceived poor hygiene standards of the staff, poor hygiene training or the preparation of foods in an unhygienic environment, with some participants particularly concerned about the likelihood of food poisoning and associated symptoms. Participants also described how low hygiene ratings would also prevent them from enjoying the food and would reflect the quality of the food provided. However, some participants noted that the food hygiene rating is only relevant where they are unfamiliar with a business, and this would not matter if they had previously ordered from the business.

## Discussion

As far as the researchers are aware, this is the first study to explore consumer knowledge and engagement with dark kitchens in a UK context. The research showed that awareness of dark kitchens was generally poor amongst UK consumers, although takeaway consumption and prevalence of takeaway purchasing was high, with over a third of consumers having takeaways at least once per week, spending around £20 per week. This is slightly higher, but comparable to the Living Costs and Food Survey ( Office for National Statistics, 2017), which suggested that the amount spent on takeaway foods and snacks consumed per household ranged from £2.80 to £12.70 per week. Most takeaways were ordered by consumers using third-party aggregator apps such as Just Eat, Deliveroo and Uber Eats or brand-specific apps to make purchases, rather than visiting the premises themselves. Whilst most consumers considered taste, quality, cost and familiarity with the business, few consumers had knowingly purchased from a dark kitchen and most would use a dark kitchen despite not being able to visit the premises. Therefore, whilst the awareness of dark kitchens was low, the prospective purchase was high which aligns with previous research conducted by Hakim *et al.* (2022) who found that consumers demonstrated an intention to buy food produced in dark kitchens, even if they did not know how to describe or define them.

In the present study, participants viewed the food hygiene rating of a food business – based on an inspection from local authority environmental health officers – as a proxy of food quality, where lower hygiene ratings are synonymous with lower quality foods. Food quality is a broad term which characterises aspects of foods that are important to governments, the food industry and consumers, including external factors (e.g., size, colour), internal factors (e.g., microbial load, foreign bodies, nutritional profile), texture and flavour ( Tanner, 2016). As such, a large proportion of the sample did not want to purchase food from a dark kitchen due to concerns related to food hygiene and cleanliness standards, the compliance with organisational, legal and regulatory frameworks. These findings align with the Food and You Survey, which reported that consumers’ most common concerns were related to food safety and hygiene (33%) and food quality (29%) ( Food Standards Agency, 2024). However, only 21% reported they had concerns with food, suggesting that the unknown nature of dark kitchens may increase consumer concern. This links with findings by Hakim *et al.*, 2022 and Cai *et al.*, 2022, who found better perceived food safety practices and trust in the food business determined consumers’ willingness to pay for food from dark kitchens. Cai *et al.* (2022) describe the link between personal and societal benefits or risks and participants’ high or low trust in a dark kitchen establishment, respectively. In the present work, more than half of the participants reported that they looked at food hygiene ratings prior to ordering food, and that the rating determined their willingness to purchase. This is higher than the 43% of respondents who reported checking food hygiene ratings of food businesses (either at the business premises or online) in the Food and You Survey ( Food Standards Agency, 2024). However, this may be due to better accessibility of information on third-party online food delivery platforms. Similar to the present findings, the Food and You Survey found consumers would not consume food from a restaurant or takeaway with a food hygiene rating of zero to two. Likewise, Dsouza and Sharma (2021) surveyed consumers in India and found that post-COVID, consumers were increasingly conscious of food safety issues and recommended that safety ratings should be published to consumers.

As many dark kitchens are new, or unknown to consumers, the trust that individuals have in ordering food from these premises is variable. As a result, most respondents wanted to know whether they were ordering from a dark kitchen or a traditional takeaway restaurant for transparency. While the location of a business is available through aggregator apps, whether the business is a dark kitchen is not currently available to consumers. Aggregator and brand-specific apps should consider including information on whether the food is prepared in a dark kitchen, alongside clear food hygiene ratings, to ensure consumers are aware of the premises in which their food is being prepared and to enable informed purchasing decisions.

### Strengths and limitations

This is the first study to understand consumer perceptions of dark kitchens in the UK. The relatively large, representative sample of adults enabled trends and views of respondents to be reported on this emerging addition to the UK foodscape and provide novel insights into consumer knowledge of and engagement with dark kitchens. Whilst surveys enable researchers to capture a lot of data quickly, further qualitative research may be useful to understand some of the consumer concerns and decision-making processes in more detail. Additionally, the Prolific platform has engaged users who complete surveys regularly. This may mean participants were more technologically-literate and had more regular access to technology than a group of consumers who would respond to a paper-based survey. Therefore, there is potential that their competence of using online apps and websites is greater than some other UK consumers meaning that accessing food online is commonplace and familiar, and this should be considered when interpreting the data.

## Conclusion

Participants frequently use takeaways and are familiar with aggregator websites and apps for ordering and tracking food purchases. However, consumers are unfamiliar with dark kitchens and are not aware of or confident in identifying which businesses are dark kitchens. In addition, trust in the food safety and hygiene standards of these businesses is variable. This results in dark kitchens being viewed in a negative light; consumers would prefer more transparency in where their foods are being prepared, and specifically whether this is being prepared in a dark kitchen, to allow for more informed decision making. As the dark kitchen sector continues to evolve, it is additionally important to capture the views of wider key stakeholders, such as those working in dark kitchens or regulating the sector.

## Ethics and consent

All procedures underwent independent review by the Sheffield Hallam University research ethics committee and the application was approved on 06 February 2024 (ethics approval number: ER61546845). All participants provided written, informed consent. Procedures adhered to the Declaration of Helsinki.

### Participants

Participants were healthy adults (18 years of age or older) living in the UK. A representative sample of the national population, based on sex, age and ethnicity was recruited through the Prolific platform. The study aimed to recruit a minimum of 2,000 participants.

## Data Availability

Supporting documents are accessible for review via the Open Science Framework (OSF). Open Science Framework: NIHR Dark Kitchens. https://doi.org/10.17605/OSF.IO/6SWBK ( Beaumont, 2024). This project contains the following data: NIHR160326 WP1 Dataset (the anonymised/deidentified raw data, prior to data analysis). NIHR160326 WP1 Information Sheet (a copy of the information sheet shared with participants). NIHR160326 WP1 Consent Form (an example consent form signed by participants). NIHR160326 WP1 Qualtrics Survey (a copy of the survey completed by participants). Data are available under the terms of the Creative Commons: Attribution 4.0 (CC BY) licence. Open Science Framework: STROBE checklist for ‘Consumer knowledge of and engagement with traditional takeaway and dark kitchen food outlets’. https://doi.org/10.17605/OSF.IO/6SWBK ( Beaumont, 2024). Data are available under the terms of the Creative Commons Attribution 4.0 International license (CC-BY 4.0).
