# Supplementary material for: Consumer knowledge of and engagement with traditional takeaway and dark kitchen food outlets
Source: NIHR Open Res. 2025 Mar 10;4:64. Originally published 2024 Oct 18. [Version 2] doi: 10.3310/nihropenres.13735.2 (PMC11920686; doi:10.3310/nihropenres.13735.2)
Supplement: Peer review history

# Reviewer report: Reviewer response for version 2

**DOI:** 10.3310/nihropenres.15095.r34896

**Published:** 2025-03-18

**Authors:** Jean Adams

**Affiliations:** 1 University of Cambridge, Cambridge, England, UK

Thanks for responding to my previous comments. I have no further comments.

Is the work clearly and accurately presented and does it cite the current literature?

Partly

If applicable, is the statistical analysis and its interpretation appropriate?

Yes

Are all the source data underlying the results available to ensure full reproducibility?

Yes

Is the study design appropriate and is the work technically sound?

Yes

Are the conclusions drawn adequately supported by the results?

Partly

Are sufficient details of methods and analysis provided to allow replication by others?

Yes

Reviewer Expertise:

Diet & public health, population interventions, natural experimental evaluations

I confirm that I have read this submission and believe that I have an appropriate level of expertise to confirm that it is of an acceptable scientific standard.

# Reviewer report: Reviewer response for version 1

**DOI:** 10.3310/nihropenres.14917.r33549

**Published:** 2024-11-22

**Authors:** Purvi Mathur

**Affiliations:** 1 MANAGEMENT, Birla Institute of Technology (Ringgold ID: 28698), Ranchi, Jharkhand, India; 2 The ICFAI University, Jaipur, India

The study as it is based on the project which is undergoing in context to cloud kitchen. There are following which are required to be added:

 1.Theory need to be included. For instance the theory of consumption value is find to be appropriate for the study.

 2. Add more current references as the topic is more prominent and lots of study is already undergoing or done.

 3. Advice to refer a research paper related to your field on the Emerald Insight on International Journal of Contemporary Hospitality Management titled as 

 Consumer attitudes toward ordering from cloud kitchens: a gender and marital status perspective

 Add these suggestions and incorporate changes will actually make the study more effective.

Is the work clearly and accurately presented and does it cite the current literature?

Partly

If applicable, is the statistical analysis and its interpretation appropriate?

Partly

Are all the source data underlying the results available to ensure full reproducibility?

Yes

Is the study design appropriate and is the work technically sound?

Yes

Are the conclusions drawn adequately supported by the results?

Yes

Are sufficient details of methods and analysis provided to allow replication by others?

Yes

Reviewer Expertise:

Supply Chain, Technology Management, Consumer Behaviour

I confirm that I have read this submission and believe that I have an appropriate level of expertise to confirm that it is of an acceptable scientific standard.

## References

[1] : Consumer attitudes toward ordering from cloud kitchens: a gender and marital status perspective. *International Journal of Contemporary Hospitality Management* .2023;35(5) : 10.1108/IJCHM-03-2022-0310 1859-1879 10.1108/IJCHM-03-2022-0310

[2] : Consumer Purchase Intention and Behavior Toward Cloud Kitchen (Pandemic Opportunity) with Reference to India: An Empirical Examination. *Jindal Journal of Business Research* .2023;12(2) : 10.1177/22786821231166790 194-208 10.1177/22786821231166790

# Response

**Published:** 2025-02-06

**Authors:** Jordan Beaumont

**Affiliations:** Sheffield Hallam University, Sheffield, England, UK

**Reviewer Comment: **Theory need to be included. For instance the theory of consumption value is find to be appropriate for the study.

**Author Response: **While the theory of consumption value provides a useful framework to understand the reasons behind consumer choice, this was not considered when designing the study. Instead, we focussed on an exploratory approach. The data from our studies does not align well with this theory, and it feels erroneous to restructure the study around such theory.

 ---------------------------

**Reviewer Comment: **Add more current references as the topic is more prominent and lots of study is already undergoing or done.

**Author Response: **While we acknowledge the relative contemporary nature of this research, we believe the articles cited are up-to-date and published within the last 4 years. There is a lack of appropriate evidence when considering consumer engagement within a UK context (hence conducting our study), and while there are some studies from a global perspective, the local food environment is an important factor in this study.

 ---------------------------

**Reviewer Comment: **Advice to refer a research paper related to your field on the Emerald Insight on  International Journal of Contemporary Hospitality Management titled as

 Consumer attitudes toward ordering from cloud kitchens: a gender and marital status perspective

**Author Response: **We have now cited this work in the introduction, which now reads:

 “While there is some evidence on consumer attitudes towards dark kitchens (e.g., Pookulangara et al. (2023)),  there is limited understanding of consumer awareness of dark kitchens and how the dark kitchen sector influences consumer decision making and purchase behaviours.”

# Reviewer report: Reviewer response for version 1

**DOI:** 10.3310/nihropenres.14917.r33230

**Published:** 2024-11-07

**Authors:** Jean Adams

**Affiliations:** 1 University of Cambridge, Cambridge, England, UK

This is an interesting paper on an emerging topic.

 My key concern is the representativeness of participants and hence to whom findings are generalizable and how definitive the conclusions can be. Although it is claimed that participants are representative of the population in terms of age, sex and ethnicity no clear comparative data supporting this claim is cited. There is also no comment made on representativeness according to socio-economic position. Some acknowledgement in the discussion is made that participants may have been more technologically literate that the wider population, but the implications of this for how data should be interpreted are not made clear.

 There are also very few comparisons drawn to other data on takeaway purchasing that could also help with determining how representative the sample is. For example, how does the 40% purchasing takeaway food at least weekly and £20 weekly spend compare to data from Food & You, National Diet & Nutrition Survey, and Living Costs and Food Survey? Food and You also reports concerns with food hygiene and this comparison is also not drawn.

 It is not clear how the content of the survey was derived – both in terms of the question stems asked and the response options available. Related, there is no discussion of the validity of the questions asked.

 Food hygiene is indicated as a proxy for food quality, but there is little justification for this or what the definition of ‘quality’ is in this context. For instances, ‘food quality’ is often used to mean nutritional content, but I don’t think food hygiene is a good proxy for nutritional content.

 It would be helpful to give some indication of whether food hygiene ratings are currently available on food ordering apps.

 There are some inconsistencies in the text – for instance:

1.  it’s claimed in the Plain Language Summary that dark kitchens are very common in the UK, but then noted in the introduction and discussion that it’s not clear how common they are.
2. The aims in the abstract focus only on awareness and engagement with dark kitchens, whereas the main text aims additionally include traditional kitchens. Indeed, whilst the abstract and introduction frame the paper in terms of dark kitchens, the title is more inclusive and it seems very few questions in the survey were specific to dark kitchens
3. It’s stated in the text that 40.0% purchased takeaway at least weekly, but in table 3 the figures are 0.1+0.3+2.4+34.3 = 37.1%
4. Findings related to concern with hygiene are presented in the abstract as specific to dark kitchens, but in table 6 they seem more generic to any takeaway purchasing

There are some other minor slips with referencing – for instance:

1. Keeble et al 2023 posit that increasing popularity of dark kitchens may have occurred during the covid-19 pandemic, not that they did
2. Keeble et al 2020 indicate that the nutritional quality of food available through online delivery services “remains unclear” – not that it is “low”

Some numbers in the tables don’t add up and other details are missing. All tables should be checked carefully – for instance:

1. Student status % don’t sum to 100 in Table 1
2. It’s not clear in Table 2 if income per week, month, year or if it’s adjusted for household composition
3. It’s not clear in Table 4 if spend is per individual, per household, or something else.

Is the work clearly and accurately presented and does it cite the current literature?

Partly

If applicable, is the statistical analysis and its interpretation appropriate?

Yes

Are all the source data underlying the results available to ensure full reproducibility?

Yes

Is the study design appropriate and is the work technically sound?

Yes

Are the conclusions drawn adequately supported by the results?

Partly

Are sufficient details of methods and analysis provided to allow replication by others?

Yes

Reviewer Expertise:

Diet & public health, population interventions, natural experimental evaluations

I confirm that I have read this submission and believe that I have an appropriate level of expertise to confirm that it is of an acceptable scientific standard, however I have significant reservations, as outlined above.

# Response

**Published:** 2025-02-06

**Authors:** Jordan Beaumont

**Affiliations:** Sheffield Hallam University, Sheffield, England, UK

**Comment: **My key concern is the representativeness of participants and hence to whom findings are generalizable and how definitive the conclusions can be. Although it is claimed that participants are representative of the population in terms of age, sex and ethnicity no clear comparative data supporting this claim is cited.

**Response: **Our participant sample aligns with the latest census data; the Prolific platform takes the intended sample size and stratifies it across three demographics: age, sex and ethnicity.

 For example, the estimated number of people with gender identity different from their sex registered at birth based on census data is 0.5% compared with 0.7% in our dataset. Similarly, the current study aligns with census data for minority ethnic groups (e.g., Black, Black British, Black Welsh, Caribbean or African, Census = 4.0%, current study = 3.3%; Mixed or Multipleethnic groups, Census = 2.9%, current study = 2.5%).

 We have made this clearer in the manuscript by adding the following sentences:

 “A representative sample of the national population was recruited through the Prolific platform; sample size was stratified based on sex, age and ethnicity in line with Census data (Office for National Statistics, 2024).” (method, page 3)

 “A representative sample of the UK population were recruited, with demographic profiles (for age, sex and ethnicity) aligning with the most recent Census data (Office for National Statistics, 2024).” (results, page 4)

**Comment: **There is also no comment made on representativeness according to socio-economic position.

**Response: **We have now added in data on socioeconomic position, based on Index of Multiple Deprivation (determined using postcode data). This has been included in Table 1.

**Comment: **Some acknowledgement in the discussion is made that participants may have been more technologically literate that the wider population, but the implications of this for how data should be interpreted are not made clear.

**Response: **The following text has been amended/added to address this: “Therefore, there is potential that their competence of using online apps and websites is greater than some other UK consumers meaning that accessing food online is commonplace and familiar, and this should be considered when interpreting the data.”

**Comment: **There are also very few comparisons drawn to other data on takeaway purchasing that could also help with determining how representative the sample is. For example, how does the 40% purchasing takeaway food at least weekly and £20 weekly spend compare to data from Food & You, National Diet & Nutrition Survey, and Living Costs and Food Survey?

**Response: **We have compared our data with that from the Food and You survey, and included the following comments in the discussion (page 9) for context:

 “This is slightly higher, but comparable to the Living Costs and Food Survey (Office for National Statistics, 2017), which suggested that the amount spent on takeaway foods and snacks consumed per household ranged from £2.80 to £12.70 per week.”

 “These findings align with the Food and You Survey, which reported that consumers’ most common concerns were related to food safety and hygiene (33%) and food quality (29%) (Food Standards Agency, 2024). However, only 21% reported they had concerns with food, suggesting that the unknown nature of dark kitchens may increase consumer concern.”

**Comment: **Food and You also reports concerns with food hygiene and this comparison is also not drawn.

**Response: **We had added this comparison to the discussion (page 9), which now reads: “This is higher than the 43% of respondents who reported checking food hygiene ratings of food businesses (either at the business premises or online) in the Food and You Survey (Food Standards Agency, 2024). However, this may be due to better accessibility of information on third-party online food delivery platforms. Similar to the present findings, the Food and You Survey found consumers would not consume food from a restaurant or takeaway with a food hygiene rating of zero to two.”

**Comment: **It is not clear how the content of the survey was derived – both in terms of the question stems asked and the response options available. Related, there is no discussion of the validity of the questions ask  ed.

**Response: **The survey incorporates standardised questions (e.g., census demographic questions) with those generated by the research team in response to study aims. The team worked with PPI and stakeholder groups to produce a draft survey, which was then piloted and refined. A copy of the survey has been made available via the Open Science Framework (link in text). The following statement has been added into the method to provide clarity on this approach: “The survey was designed with input from Patient and Public Involvement (PPI) and stakeholder groups, and was refined and piloted prior to the study to ensure data collected answered the research aims.”

**Comment: **Food hygiene is indicated as a proxy for food quality, but there is little justification for this or what the definition of ‘quality’ is in this context . For instances, ‘food quality’ is often used to mean nutritional content, but I don’t think food hygiene is a good proxy for nutritional content.

**Response: **To provide further clarity, we have added the following statement to the discussion (page 9): “Food quality is a broad term which characterises aspects of foods that are important to governments, the food industry and consumers, including external factors (e.g., size, colour), internal factors (e.g., microbial load, foreign bodies, nutritional profile), texture and flavour (Tanner, 2016).”

**Comment: **It would be helpful to give some indication of whether food hygiene ratings are currently available on food ordering apps.

**Response: **This has been added on page 2, which now reads: “However, the online third-party aggregator platforms do provide a level of data transparency for the consumer by providing food hygiene ratings and other purchaser reviews on their platforms which are easy to access, to allow the consumers to make more informed food choices.”

**Comment: **There are some inconsistencies in the text – for instance:

- it’s claimed in the Plain Language Summary that dark kitchens are very common in the UK, but then noted in the introduction and discussion that it’s not clear how common they are.

**Response: **This has been amended to: “This work is important because dark kitchens are present in the UK and are a new type of food outlet which are less well-researched.”

  

- The aims in the abstract focus only on awareness and engagement with dark kitchens, whereas the main text aims additionally include traditional kitchens. Indeed, whilst the abstract and introduction frame the paper in terms of dark kitchens, the title is more inclusive and it seems very few questions in the survey were specific to dark kitchens

**Response: **This has now been amended and reads: “The present work explores consumer understanding of and engagement with dark kitchen and traditional takeaway establishments.”

  

- It’s stated in the text that 40.0% purchased takeaway at least weekly, but in table 3 the figures are 0.1+0.3+2.4+34.3 = 37.1%

**Response: **This has been corrected.

  

- Findings related to concern with hygiene are presented in the abstract as specific to dark kitchens, but in table 6 they seem more generic to any takeaway purchasing

**Response: **This has been amended and now reads: “They are a contemporary addition to the food environment in the United Kingdom (UK), possibly finding popularity during the COVID-19 pandemic (Keeble et al., 2023).”

**Comment: **There are some other minor slips with referencing – for instance:

- Keeble et al 2020 indicate that the nutritional quality of food available through online delivery services “remains unclear” – not that it is “low”

**Response: **This has been amended and now reads: “The lack of clarity in nutritional quality of foods offered by many of these businesses (Fernandez & Raine, 2021; Keeble et al., 2020), and potential for poor food safety practices (Crawford & Benjamin, 2019) are cause for concern, and best practice is yet to be established.”

  

- Keeble et al 2020 indicate that the nutritional quality of food available through online delivery services “remains unclear” – not that it is “low”

**Response: **This has been amended and now reads: “The lack of clarity in nutritional quality of foods offered by many of these businesses (Fernandez & Raine, 2021; Keeble et al., 2020), and potential for poor food safety practices (Crawford & Benjamin, 2019) are cause for concern, and best practice is yet to be established.”

**Comment: **Some numbers in the tables don’t add up and other details are missing. All tables should be checked carefully – for instance:

 1. Student status % don’t sum to 100 in Table 1

 2. It’s not clear in Table 2 if income per week, month, year or if it’s adjusted for household composition

 3. It’s not clear in Table 4 if spend is per individual, per household, or something else.

**Response: **These errors have been addressed, and all tables have been checked for errors and amended as required.
